# Thriving Beyond Adversity: A Prospective Longitudinal Cohort Study Using a Strength-Based Approach Depicts Indigenous Adolescents with Less Adverse Childhood Experiences (ACEs) Had Fewer Neurodevelopmental Disorders (NDDs)

**DOI:** 10.3390/bs14111047

**Published:** 2024-11-05

**Authors:** Md Irteja Islam, Bernadette Yan Yue Lam, Tuguy Esgin, Alexandra Martiniuk

**Affiliations:** 1 Sydney School of Public Health, Faculty of Medicine and Health, The University of Sydney, Edward Ford Building, A27 Fisher Road, Sydney, NSW 2006, Australia; blam5712@uni.sydney.edu.au (B.Y.Y.L.); t.esgin@curtin.edu.au (T.E.); alexandra.martiniuk@sydney.edu.au (A.M.); 2 Research, Innovation and Grants, Spreeha Bangladesh, Gulshan-2, Dhaka 1212, Bangladesh; 3 Centre for Health Research, The University of Southern Queensland, Darling Heights, Toowoomba, QLD 4350, Australia; 4 Dean Indigenous Engagement, Faculty of Business and Law, Curtin University, Bentley, Perth, WA 6102, Australia; 5 School of Medical and Health Sciences, Edith Cowan University, Joondalup, Perth, WA 6027, Australia; 6 Dalla Lana School of Public Health, The University of Toronto, 155 College St Room 500, Toronto, ON M5T 3M7, Canada

**Keywords:** adverse childhood experiences, neurodevelopmental disorders, ADHD, autism, intellectual disability, learning disability, adolescents, Indigenous population, strength-based analysis, longitudinal study

## Abstract

Improving social and emotional well-being (SEWB) among Indigenous adolescents is crucial. Since neurodevelopmental disorders (NDDs) are common in Indigenous people and adverse childhood experiences (ACEs) are important contributors to negative health outcomes throughout the lifespan, we investigated whether limited ACE exposure is associated with reduced risk of NDDs in Australian Indigenous teens using the data from multiple waves (Wave 1 to Wave 9, and Wave 11) of the Longitudinal Study of Indigenous Children (LSIC). We also examined the role of other protective factors, such as Indigenous cultural identity and school connectedness, against NDDs. A strengths-based approach using mixed-effects logistic regression models examined the protective effect of limited ACE exposure (from LSIC waves 1–9) on NDDs (outcome from LSIC wave 11), adjusting for sociodemographic factors. The NDDs included autism, ADHD, intellectual, neurological, and specific learning disabilities. Of the 370 individuals analysed, 73.2% valued Indigenous cultural identity, and 70.5% were strongly connected at school. More than one-fourth (27.8%) reported limited ACE exposure, while the majority was not diagnosed with NDDs (93%). Longitudinal analysis revealed limited ACE exposure was 6.01 times (95% CI: 1.26–28.61; *p* = 0.024) more likely to be protective against NDDs compared to those exposed to multiple ACEs. Moreover, valuing cultural identity (aOR = 2.81; 95% CI: 1.06–7.39; *p* = 0.038) and girls (aOR = 13.88; 95% CI: 3.06–62.84; *p* = 0.001) were protective against NDDs compared to their respective counterparts. Our findings highlight the need to prevent ACE exposure and promote Indigenous cultural identity in preventing negative health outcomes and the exacerbation of health inequities to strengthen the SEWB of Indigenous communities.

## 1. Introduction

Aboriginal and Torres Strait Islander Peoples (hereafter respectfully referred to as Indigenous) have a long and rich history of resilience, cultural identity, community spirit, family connections, and creativity [1]. These strengths have helped them to overcome many challenges, including the historical and contemporary effects of colonialism [2,3,4]. Despite these challenges [5,6], Indigenous Australians are making significant progress in improving their social and emotional well-being (SEWB). Nationwide efforts, such as Closing the Gap [7] and the National Aboriginal and Torres Strait Islander Health Plan [8], are supporting Indigenous communities to achieve their health and well-being goals. Indigenous children are often able to draw on their culture and traditions to develop unique coping mechanisms and strategies for success [9,10]. Indigenous children are also more likely to have strong family and community support networks, which can play a vital role in their development and well-being [1].

Indigenous children are resilient and resourceful, and they have many strengths that can help them overcome the challenges of neurodevelopmental disorders (NDDs) [10,11,12]. Disorders of early brain development are often called neurodevelopmental disorders that include heritable conditions such as autism. NDDs may lead to cognitive, communication, motor, or behavioural difficulties [13,14]. These include attention deficit hyperactivity disorder (ADHD) [15], intellectual disability [16], autism spectrum disorder (ASD) [16], cerebral palsy [17], learning disability [18], and conduct disorder [18]. Evidence continues to grow regarding what causes neurodevelopmental disorders, risk factors, and how we can prevent and treat these disorders.

Across childhood developmental domains, it is estimated that more than one-third (34%) of Indigenous children are meeting developmental targets, which is low compared to non-Indigenous children in Australia [19]. Adversity and NDDs have a complex interrelationship, with adversity being a risk factor for NDDs and children with NDDs more likely to experience adversity [18,20]. Being diagnosed with a NDD may mean a child is less likely to reach developmental milestones at the same time as their peers, which has lifelong implications [21,22]. Considering these long-term sequelae, researchers have sought to examine the risk factors leading to the development of NDD as well as functioning with an NDD.

A multitude of risk factors are predisposed to NDDs [18]. These range from social deprivation, infections, trauma, and genetic and environmental factors [23]. For instance, perinatal factors (e.g., prematurity, low birth weight, respiratory distress), which are more prevalent in Indigenous populations [24], have been demonstrated to have associations with the child later receiving diagnoses of ASD and/or ADHD [25]. Maternal factors such as younger age, lower education status, smoking, alcohol, and substance use may lead to poor antenatal care and may increase the risk for subsequent NDDs [13,26]. All these risks are further exacerbated by remoteness, socioeconomic disadvantages, and culturally inappropriate services [26]. Promisingly, Indigenous mothers who ceased smoking and had cultural-based resilience had infants with a lower risk of a broad range of adverse health outcomes [27]. Prevalence data reassuringly reflects this, as 89% of Indigenous Australian newborns are of healthy birthweight, and smoking rates in pregnant women have dropped from 52% to 44% [19]. Culturally safe antenatal care not only considers the historical context of Indigenous mothers but is thought to be of equal importance to physical care [28].

Adverse childhood experiences (ACEs) are intense and recurring sources of stress a child may be exposed to before the age of 18 years [29] can be categorised into domains of abuse, neglect, household or family dysfunction/instability or family poor mental health, and violence [30]. Stress from ACEs can be toxic and can impact brain development. ACEs also increase the risk of chronic illness and mental illness later in life [31]. However, ACEs can be prevented. Though suffering an ACE does not discriminate [32], experiencing ACEs is notably elevated in Indigenous Australians due to historical trauma and continued discrimination [2,3,4]. Indigenous adolescents are more likely than their non-Indigenous peers to report ≥3 major negative life events (67% vs. 14%) and multiple adversities (22% vs. 8%) [33]. It was found that 96% of Indigenous children experienced ≥1 ACE by 8 years and accumulated ≥6 risk factors for mental illnesses by early adolescence [33]. ACEs negatively, in a dose response relationship, affect a broad range of health outcomes in all contexts and populations, as documented in several systematic reviews [3,34,35]. Among Australian youth, parental incarceration and domestic violence were found to predict higher rates of conduct disorder [36], whereas physical or sexual abuse predicted self-harming behaviour [37]. Similarly, cumulative ACEs were found to be positively associated with ASD, ADHD, intellectual disability, and learning disability among non-Indigenous American children [38]. Yet, ACEs do not always lead to negative outcomes [39,40]. Some Indigenous young people who have been bullied exhibit resilience that renders them better able to tackle future life challenges; this resilience is afforded by a sense of community belonging that lowers distress and buffers the impact of bullying [12]. Despite exposure to ACEs, Indigenous Australians are extremely resilient [1,10].

Most of the current literature on ACEs and NDDs comes from cross-sectional studies and is focused on adults [41,42,43,44] with less evidence in paediatric populations [45], and even less in Indigenous communities [3,33]. Given the pervasive deficit discourse in Indigenous research, we strive to use a strengths-based approach to positively reinforce Indigenous perspectives and decolonise the narrative as a starting point from which to improve health inequities [2,46,47]. To our knowledge, this prospective cohort study is the first that aimed to investigate whether limited exposure to ACEs is associated with reduced likelihood of neurodevelopmental disorders among Indigenous Australian adolescents. This study additionally examined the role of potential protective factors (i.e., Indigenous cultural identity, school connectedness) in reducing the incidence of health outcomes, such as NDDs, among Australian Indigenous adolescents.

## 2. Methods

### 2.1. Data Source and Study Design

Data from ‘Footprints in Time: Longitudinal Study of Indigenous Children (LSIC)’, an ongoing nationwide prospective longitudinal cohort study funded by the Australian Government Department of Social Services (AGDSS) in Australia, was used for secondary data analysis [48]. The LSIC study provides an opportunity to examine early life factors contributing to the health and development of Indigenous youth over time, with the overall aim of the study to better understand relationships to make policy and program changes to ultimately close the gap on health disparities and challenging life circumstances disproportionately faced by Indigenous Australians [49]. The following research questions underscore the objective of the overarching LSIC study: “What do Aboriginal and Torres Strait Islander children need to have the best start in life to grow up strong; … to stay on track or become healthier, more positive, and strong; … the importance of family, extended family, and community in the early years of life and when growing up?” [49]. To emulate these sentiments, a strengths-based approach, initially pioneered by Thurber, Thandrayen [47], was used in this study, enabling the telling of a positive story that best reflects Australian Indigenous community values. More information regarding the LSIC study design has previously been outlined elsewhere [48,49,50].

### 2.2. Participants and Sample Selection

**Figure 1:**
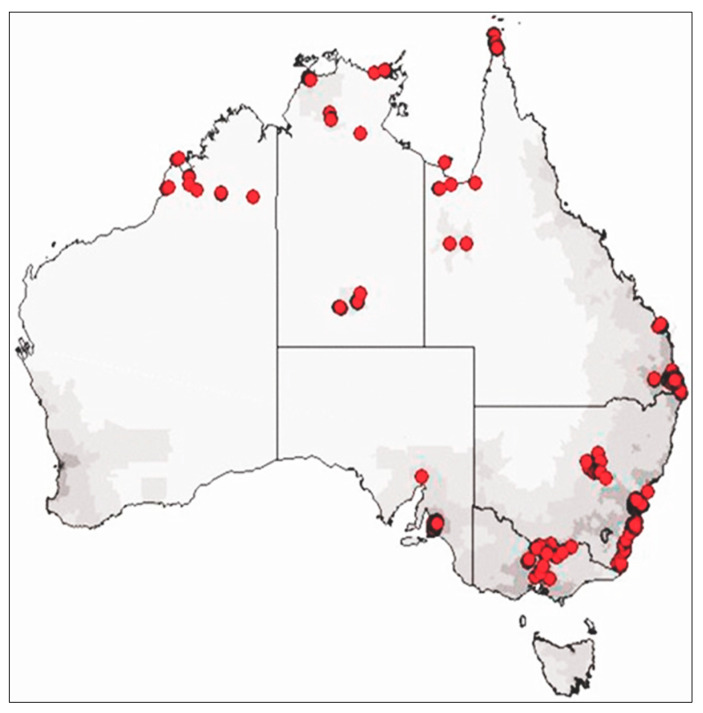
Geographic distribution of the LSIC sample population in Australian map in red dots.

Using a two-stage non-random purposive clustered sampling design by LSIC, 150 indigenous families were interviewed at each of the 11 sites across the country, varying from urban, regional, and remote locations [48], as depicted in Figure 1. The baseline survey (i.e., Wave 1) of LSIC commenced in 2008 with two distinct cohorts of Indigenous children: B-cohort (0.5 to 2.0 years) and K-cohort (3.5 to 5.0 years) at the baseline survey. In Wave 1 (in 2008), 1671 children were enrolled, and these same children continued to participate in subsequent waves of data collection in each year, though the sample retention decreased to 71.2% by Wave 11 (in 2018). The annual collection of data was via face-to-face interviews with the interviewer and respondent (e.g., parents, study children, teachers). Voluntary written informed consent by the parent was obtained before each wave of study [49].

**Figure 2:**
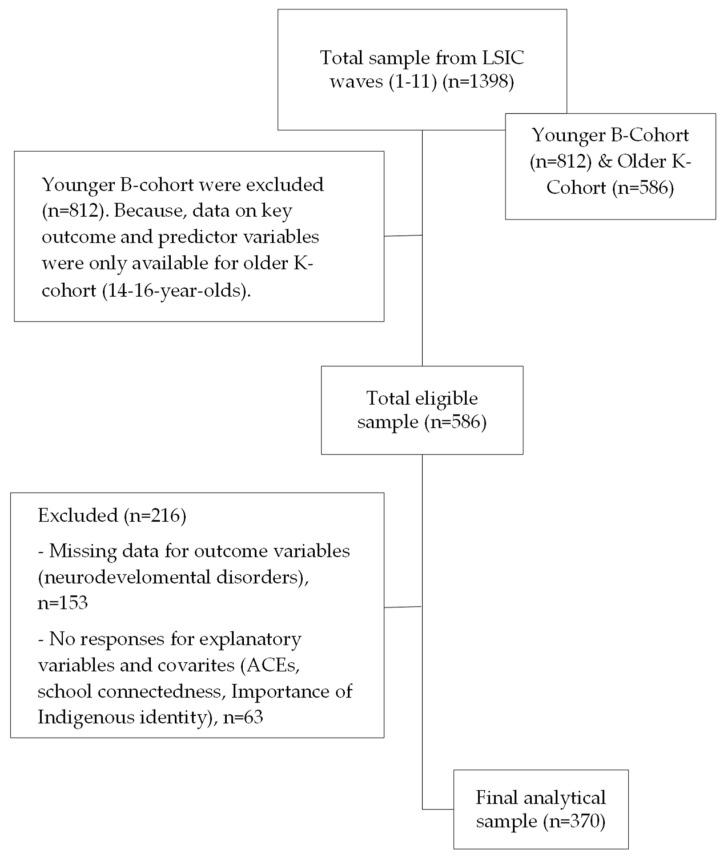
Flow chart for analytical sample selection.

The current study used data from 10 longitudinal LSIC waves (Wave 1–Wave 9, and Wave 11) conducted between 2008 and 2018 involving the same participants linked between waves using unique identifier numbers to incorporate 370 K-cohorts from the LSIC database. Including the K-cohort ensured the analysis of participants with complete data on the outcome variables (i.e., NDDs) in Wave 11 (2018), which chronologically followed exposure variables (ACEs, Indigenous cultural identity, school connectedness) in Waves 1 (2008) to 9 (2016) (*n* = 370). Participants who did not respond to either outcome or predictor variables were excluded from statistical analyses (*n* = 216). The flowchart below (Figure 2) demonstrates the analytical sample selection process.

### 2.3. Measures

Variables determined from the previous literature to be associated with ACEs and NDDs that were relevant to Indigenous populations were examined [2,3,33,38,51,52]. The selection of sociodemographic covariates was made following a strengths-based approach, a method that builds upon the traditional protective factors approach and diverts focus towards positive attributes rather than a deficit narrative [47]. This ‘Positive Outcome Approach’ [47] measures the association between positive factors (e.g., limited exposure to ACEs), as well as positive factors such as Indigenous cultural identity, feeling connected to family and school, and positive outcome variables (e.g., no NDDs) rather than using risk factors (e.g., exposure to ACEs) and adverse outcome variables (e.g., poor physical and mental health). All variables included are listed in Table 1.

### 2.4. Cultural Integrity

This study allowed Indigenous and non-Indigenous authors to learn from one another. It allowed the Noongar/Yamatji Aboriginal co-author to expand his research capacity, and the research benefitted from his leadership, expertise, and understanding of Indigenous knowledge. It also gave him the ability to govern, share, maintain, and enhance his cultural and intellectual legacy by steering the research processes with Indigenous ways of knowing, being, and doing. Our Canadian co-author also contributed her perspective, having Canadian First Nations and Inuit family, along with Ukrainian heritage. Although the study did not employ an Indigenous research paradigm due to its quantitative nature, it was influenced by a strength-based model and incorporated aspects of the CREATE Aboriginal and Torres Strait Islander quality appraisal tool to ensure cultural integrity [53].

### 2.5. Statistical Analysis

All statistical analyses were performed using Stata v14.1 (Stata Corporation, College Station, TX, USA). For descriptive analyses, we ran frequency (*n*) and percentage (%) tests on outcome, explanatory, and sociodemographic variables. For multivariate analyses, given the nested nature of the study sample (i.e., Indigenous adolescents aged 14 to 16 years) within households and households within clusters (i.e., geographic area based on Level of Relative Isolation classification), we used mixed-effect logistic regression models (as recommended by the LSIC guidelines) to estimate the odds ratio (OR) for the impact of limited exposure to ACEs and positive variables (i.e., Indigenous cultural identity, school connectedness) on NDDs. Bivariate associations between each predictor variable and outcome variables were examined by estimating unadjusted ORs. Variables yielding a *p*-value of <0.05 in the unadjusted models were then included in the adjusted model. Multivariable analyses examined ACEs as the main explanatory variable while adjusting for potential covariates.

### 2.6. Ethics Approval

The LSIC study was ethically approved by the Australian Government Department of Health Departmental Ethics Committee from 1 January 2006 to 30 June 2018, and, since 1 July 2018, LSIC has been approved by the Human Research Ethics Committee of the Australian Institute of Aboriginal and Torres Strait Islander (ethics code: AIATSIS). The authorship team obtained LSIC data access approval on 19 July 2023 from the National Centre of Longitudinal Data and the Australian Data Archive for this research project using LSIC data (Application Reference No. 510943). The nature of this secondary data is consistent with ‘Outcome A’ of the University of Sydney Research Ethics Board and did not require ethical approval from the University of Sydney.

## 3. Results

Descriptive data of the study population are presented in Table 2. Our final study sample included 370 Indigenous adolescents, with complete data on variables of interest, including our outcome variables, main explanatory variable, and covariates. Adolescents’ ages ranged from 14 to 16 years, with a mean (SD) age of 15.05 (0.44) years. There was a 1.056:1 male-to-female ratio. Adolescents largely resided in regional/remote Australia (72.2%), where nearly half of the respondents (44%) were considered socioeconomically advantaged (i.e., Q4 and Q5 on the IRSEO index, explained in detail in Table 2). Almost three-quarters emphasised the importance of their Indigenous cultural identity (73.2%) and felt strongly connected at school (70.5%). Experiencing limited ACEs (one or no) comprised 27.8% of the study sample. Moreover, there was a high proportion of Indigenous adolescents who did not report a diagnosis of an NDD (No, 93.0% vs. Yes, 7%) in the sample.

The results from the mixed-effect logistic regression models are portrayed in Table 3. The adjusted model in the longitudinal analysis shows that Indigenous adolescents with limited exposure to ACEs were six times (95% CI: 1.26–28.61; *p* = 0.024) less likely to have NDDs compared to those who reported multiple ACEs. Girls were 13.88 times (95% CI: 3.06–62.84; *p* = 0.001) less likely to report having a NDD compared to boys. Indigenous adolescents who valued Indigenous cultural identity (95% CI: 1.06–7.39; *p* = 0.038) were 2.81 times less likely to report having an NDD compared to those who did not find Indigenous cultural identity important.

## 4. Discussion

Highlighting progress instead of discrepancies not only improves health and well-being outcomes but also empowers, encourages continual improvements, and shifts the power dynamic back to Indigenous communities [47,54]. Following a strength-based approach, this study found that Indigenous adolescents who experienced fewer ACEs were female, and those who valued their Indigenous cultural identity were less likely to report having an NDD compared to those who had more exposure to ACEs. These results highlight the importance of preventing early exposure to ACEs to allow for optimal health outcomes in Indigenous adolescents. Elucidating the cultural and protective factors that allow us to do so deepens our understanding of the holistic needs of Indigenous children. While reducing exposure to ACEs is valuable for all children, the relationship between ACEs and NDDs is complex. For example, parents with an NDD may be more likely to live in adverse and stressful circumstances; at least some NDDs have known heritable fractions.

We revealed that limited exposure to ACEs was significantly associated with less risk of NDDs in Indigenous adolescents aged 14–16 years. This is consistent with previous studies examining the cumulative effect of multiple ACEs on NDDs among adults [34,55] and non-Indigenous children [38,56,57]. One of the possible explanations could be the neurobiological mechanism by which repeated exposure to ACEs causes chronic stress activation, causing neuroendocrine, immune, and metabolic disruptions, which underpin disease development [58]. It is therefore feasible that Indigenous adolescents who have faced continual psychosocial stresses are unduly affected [59]. For instance, racism and bullying have been heavily studied in Indigenous children as risk factors for poor social-emotional well-being and socioemotional problems, including hyperactivity [33,60]. Exposure to threat, deprivation, abuse, and neglect has also been shown to reduce brain volume and dysregulate hormonal and metabolic systems, which hinders childhood development [61,62]. Additionally, ‘The Protective Factors Framework’ suggests that parental attributes such as nurturing, adequate parenting knowledge, resilience, and a supportive social network may contribute to social and emotional competence in children, thus decreasing the risk of NDDs [63]. Moreover, epigenetics provides another mechanism relevant to Indigenous communities, as it describes the environmental modification of genes without altering genes but affects how genes work [3,64] through intergenerational or parental exposure to ACEs and other environmental factors [65]. For example, maternal childhood maltreatment has been shown to predict offspring NDDs (e.g., ASD, ADHD, intellectual disabilities, cerebral palsy) [66,67]. However, a study conducted in the USA found that ACEs were not observed to increase the risk of neurodevelopmental delays in children aged between 2 and 11 years [68]. The authors hypothesised that perhaps the adverse experiences affect development over time, creating an opportunity for early intervention and perhaps explaining why our study of older youth found a relationship between ACEs and NDDs but Mehari et al. [68] did not.

Furthermore, the findings of our study indicated that girls were less likely to develop NDDs compared to boys. Historically, it has been reported that NDDs are skewed towards boys [51,69,70,71] and boys who experience ACEs are more susceptible to NDDs than girls [51,72]. Increased male susceptibility to ACE exposure may be explained by evolutionary differences in the neural and epigenetic mechanisms between males and females, which manifest in different sensitivities to ACE exposure [51,73]. For instance, a systematic review hypothesised that autistic girls possess higher sensitivities to recognise socially salient stimuli and have richer language expression, which may render them better able to ‘camouflage’ their deficits in socializing or communicating [74,75]. Moreover, there may be a function of society diagnosing more boys due to a preponderance of externalising behaviours versus girls who tend toward more internalising behaviours, as well as gender differences in learnt coping strategies and gender stereotypes in females that may be playing a role rather than a true gender difference [75].

In addition, this study found that holding Indigenous cultural identity in high regard has a protective effect against NDDs. Cultural identity’s protective effect has been evidenced in Indigenous populations against allostatic load [61], poor mental health conditions [3,36], and social and emotional and hyperactivity problems [76]. This may be because cultural identity increases resilience or self-esteem and boosts Indigenous adolescents’ ability to navigate racial discrimination through increased cultural engagement, connection to the community, and strengthened bonds with family and kin [77,78]. Another study reported that cultural identity directly contributes to resilience [46], equipping children and adolescents with the tools to ensure good neurodevelopment and psychosocial health [10]. Results from previous studies show that Indigenous Australian children exposed to ACEs possess a higher level of resilience stemming from positive self-identity, which could improve social-emotional well-being [33,79] and potentially reduce the chance of developing NDDs. It has been reported that cultural identity is ever-evolving and context-bound, the formation of which is a critical rite of passage for adolescent development and an indicator of well-being and resilience universally [80]. Cultural identity can be strengthened through parents’ sharing of cultural knowledge [46] by relating their ancestral heritage, relying on social support systems and interpersonal relationships to reinforce a sense of Indigeneity, which subsequently empowers individual and family social-emotional well-being [81,82]. A systematic review examining ACEs within Indigenous populations stated an evidence gap regarding examining ACEs within Indigenous populations and found that those with a high exposure to ACEs found cultural engagement to be less appealing or useful [3].

## 5. Strengths and Limitations

A major strength of this study was its longitudinal study design and population (i.e., Indigenous adolescents). Prospective cohort studies establish a clear temporal sequence between exposure to childhood traumas and the development of neurodevelopmental conditions. This enables research to ascertain the timing of exposure and subsequent outcomes, strengthening the ability to establish causality. ACEs are highly unlikely to be the sole exposures of importance upon risk for developmental disorders, and genetics and other environmental factors also feature. Nonetheless, this study has its limitations. This study had a small sample size (*n* = 370) due to non-response from participants. A legacy of the historical trauma of the Stolen Generation is the removal and institutionalisation of children, which may cause fear or reluctance to engage with the healthcare system [83]. As such, undiagnosed children may not be captured by the outcomes of NDD in the LSIC survey. Although not nationally representative, data were purposefully collected across 11 sites to reflect the socioeconomic and community distribution of Indigenous populations in Australia. Moreover, using self-reports may have caused recall bias, as remembering past ACEs may be less reliable if time has elapsed. Health conditions may have been potentially misclassified as diagnoses were self-reported. Social desirability bias was possible given the stigma surrounding ACEs, which may have led parents to underreport events. Furthermore, this study could not include all the standard ACE categories listed by the World Health Organization [29], as items regarding physical and/or sexual abuse/neglect, mental illness, and war-related variables for the Indigenous parents/children were not available in the dataset. In addition, dichotomising ACEs into a binary variable potentially oversimplifies and homogenises all adversities [34]. Lastly, this study adopted a strength-based approach, which challenged the deficit-based narrative that often surrounds Indigenous children. The strength-based approach was used with a thought to empower Indigenous children and families, as it may help to shift the focus from what is wrong with the Indigenous population to what is right with them.

## 6. Conclusions

This study found Indigenous adolescents who had less exposure to ACEs were less likely to develop NDDs, though a considerable proportion of adolescents reported multiple ACEs and only a small number had NDDs. Furthermore, the current study revealed that valuing Indigenous cultural identity has a protective effect against NDDs in Indigenous Australian adolescents. Continued longitudinal research can expand on this by examining the individual effect size of each ACE and determining which ones tend to co-occur. Further elucidation of Indigenous-specific protective factors against NDDs in youth exposed to ACEs highlights Indigenous strengths, a crucial element in shifting away from the deficit narrative, and a palatable method of giving power back to Indigenous communities. Furthermore, this would inform early intervention programs occurring from the bottom up (i.e., home, school, community, institutions, and governments) using trauma-informed care tailored to Indigenous youth, which provides a safe space that prevents re-traumatization. Additionally, we suggest a greater focus on addressing parental ACEs through positive parenting and family environments, and antenatal care for Indigenous mothers could help intercept the intergenerational transmission of adversity. Moreover, we recommend that an increased understanding of the cultural context of ACEs and NDDs will help ensure culturally safe services and support further funding for cultural activities to bolster SEWB.

## Figures and Tables

**Table 1:** List of variables.

Variables ^1^	Description of Variables
Outcome variables	
Neurodevelopmental disorders	Whether the study child was diagnosed with any neurodevelopmental disorders such as autism, ADHD, intellectual, neurological, psychiatric, and specific learning disabilities. Response options were categorised as ‘Yes’ (coded 0) and ‘No’ (coded 1).
Main explanatory variable	
Adverse childhood experiences (ACEs)	We created a composite score from five types of ACEs (highest score 5 and lowest score 0) and then categorised them into two: study children experienced 3–5 ACEs termed as ‘multiple ACEs’ (coded 0), while children with zero/one ACE termed as ‘No/Limited ACE’ (coded 1). The included ACEs were: biological parents had split up, family violence, parents/close family members mugged/robbed/assaulted, parents/close family members arrested/jailed, and study child bullied because of Indigeneity.
Covariates	
Age	Age was used as a continuous variable
Sex	The sex of the adolescents was categorised into ‘Boys’ (coded as 0) and ‘Girls’ (coded as 1).
Area of residence	The Australian Statistical Geography Standard (ASGS) classifies Remoteness Areas into five categories of relative remoteness across the country: Major Cities of Australia, Inner Regional Australia, Outer Regional Australia, Remote Australia, and Very Remote Australia [37]. From the responses, we created a binary variable, ‘Area of Residence’: ‘major cities’ were coded as ‘1’, and ‘inner regional’, ‘outer regional’, ‘remote’, and ‘very remote’ were combined as ‘regional/remote’ (coded as 0).
IRSEO index	The Indigenous Relative Socioeconomic Outcomes (IRSEO) index is comprised of socioeconomic outcomes (i.e., employment, education, income, and housing) and is used to estimate the socioeconomic status of Indigenous Australians living in each Indigenous area in Australia. The lowest IRSEO index (Quintile 1, 0–20%) signifies the most disadvantaged, and the highest IRSEO index (Quintile 5, 80–100%) indicates the most advantaged at the Indigenous area level. In this study, we categorised the IRSEO index into three: disadvantaged (quintile 1 + 2), average (quintile 3), and advantaged group (quintile 3 + 4).
School connectedness	Categorised into two categories: ‘Not much connected’ (coded as 0) and ‘Strongly connected’ (coded as 1). School connectedness was measured by whether the study child was good at school, made friends easily at school, did schoolwork, felt strong at school, and knew where/when to go at school.
Indigenous identity	Categorised into ‘Not so important’ (coded as 0) and ‘Important’ (coded as 1). According to the National Strategic Framework of Health for Indigenous Australians, Indigenous identity is one of the vital components of SEWB.

^1^ We specifically sought to include variables previously shown or hypothesised to be associated with strong social and emotional well-being for Indigenous people. Variables were also coded to be strengths-based to examine each variable as a protective factor.

**Table 2:** Descriptive statistics.

	*n* (370)	%
Age ^1^	Mean = 15.05, SD = 0.44	
Gender		
Boys	190	51.4
Girls	180	48.7
Area of residence		
Regional/Remote	267	72.2
Urban	103	27.8
Indigenous socioeconomic status		
Disadvantaged	111	30.0
Average	96	26.0
Advantaged	163	44.1
School connectedness		
Not much connected	109	29.5
Strongly connected	261	70.5
Indigenous cultural identity		
Not important	99	26.8
Important	271	73.2
Main explanatory variable		
ACEs		
Multiple (2 or more ACEs)	267	72.2
Limited (0 to 1 ACE)	103	27.8
Outcome variable		
Neurodevelopmental disorders (NDDs)		
Yes	26	7.0
No	344	93.0

^1^ Continuous variable. SD = Standard Deviation; ACE = Adverse Childhood Experience.

**Table 3:** Impact of ACEs on NDDs among Indigenous adolescents (mixed-effect logistic regression models following a strengths-based approach).

	Neurodevelopmental Disorders (NDDs)	
	Unadjusted OR (95% CI)	Adjusted ^#^ OR (95% CI)
ACEs		
Multiple (2 or more ACEs)	Ref.	Ref.
Limited (0 to 1 ACE)	5.22 * (1.18, 23.09)	6.01 * (1.26, 28.61)
Age		
Mean	1.07 (0.36, 3.20)	-
Gender		
Boys	Ref.	Ref.
Girls	12.99 ** (2.99, 56.31)	13.88 ** (3.06, 62.84)
Area of residence		
Regional/Remote	Ref.	-
Urban	0.97 (0.32, 2.95)	-
Indigenous socioeconomic status		
Disadvantaged	Ref.	-
Average	0.58 (0.12, 2.74)	-
Advantaged	1.28 (0.27, 6.03)	-
School connectedness		
Not much connected	Ref.	-
Strongly connected	2.78 (0.95, 8.10)	-
Indigenous cultural identity		
Not important	Ref.	Ref.
Important	2.80 * (1.16, 6.75)	2.81 * (1.06, 7.39)

Level of significance: * *p* < 0.05, ** *p* < 0.01; OR: Odds ratio; CI: Confidence interval. ^#^ Variables that yielded statistically significant association with the outcome (i.e., NDDs) in unadjusted models were included in the adjusted model.

## Data Availability

The LSIC dataset is publicly available from the National Centre for Longitudinal Data (NCLD) Dataverse and Australian Data Archive (ADA) on request available at https://dataverse.ada.edu.au/dataverse/lsic (accessed on 19 July 2023). Authors do not have permission to share this unit record data without endorsement from the Department of Social Services and Australian Institute of Family Studies.
